# Supplementary material for: RNA-binding protein ZFP36L1 regulates osteoarthritis by modulating members of the heat shock protein 70 family
Source: Nat Commun. 2019 Jan 8;10:77. doi: 10.1038/s41467-018-08035-7 (PMC6325149; doi:10.1038/s41467-018-08035-7)
Supplement: Supplementary file 1 — Supplementary Information [file 41467_2018_8035_MOESM1_ESM.pdf]

Supplementary Information

RNA-binding protein ZFP36L1 regulates osteoarthritis by modulating members of the heat shock protein 70 family

by Young-Ok Son et al.

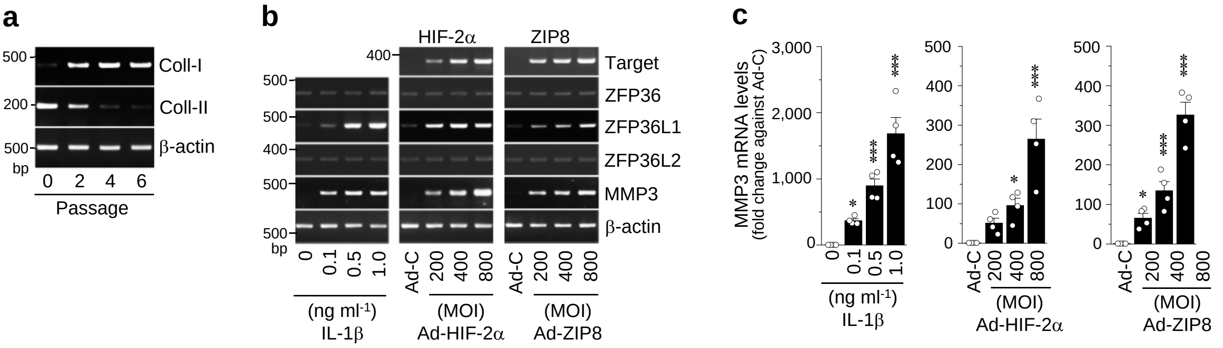

Supplementary Fig. 1. Upregulation of ZFP36L1 in chondrocytes stimulated by OA-associated catabolic signaling. (**a**) Chondrocytes were sub-cultured up to passage 6. Presented are representative RT-PCR images of type I collagen (Coll-I) and type II collagen (Coll-II) in chondrocytes of the indicated passages (*n* = 4). (**b**, **c**) Chondrocytes were treated with IL-1β for 36 hours or infected with 800 MOI of Ad-C or the indicated MOIs of Ad-HIF-2α or Ad-ZIP8 for 36 hours. The indicated mRNAs were detected by RT-PCR (**b**), and the mRNA levels of MMP3 were quantified by qRT-PCR analysis (**c**, *n* = 4). Values are presented as means ± s.e.m. (*$P < 0.05$, **$P < 0.005$, and ***$P < 0.0005$; one-way ANOVA).

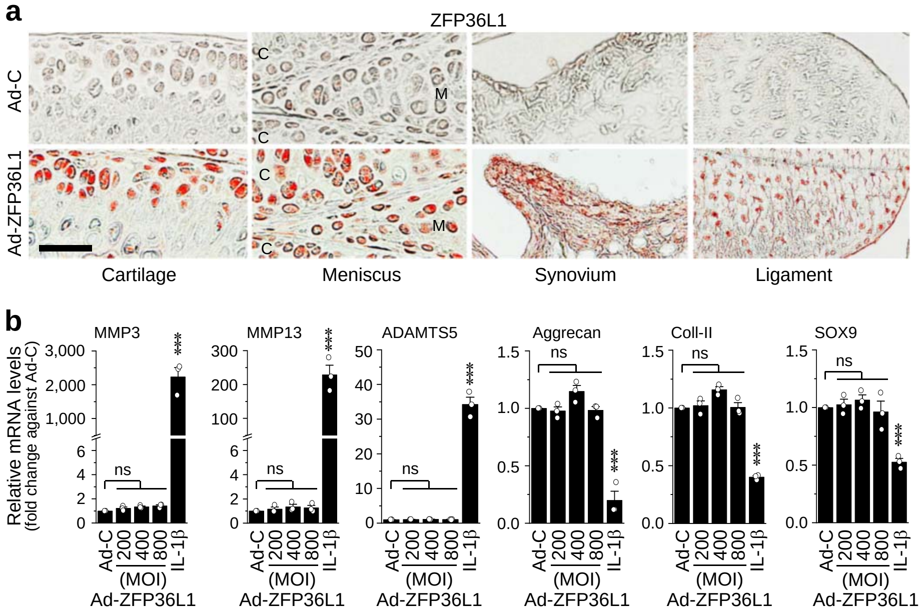

Supplementary Fig. 2. Overexpression of ZFP36L1 in joints tissues of mice IA-injected with Ad-ZFP36L1. (**a**) Mice were IA injected with Ad-C as a control or Ad-ZFP36L1. ZFP36L1 protein was detected by immunostaining of the indicated tissues. Representative images are presented (n = 5 mice per group). (**b**) RT-PCR analyses (n = 3) of indicated molecules in chondrocytes infected with 800 MOI of Ad-C or the indicated MOI of Ad-ZFP36L1. IL-1β (1 ng ml^{-1}, 36 hours) was used as a positive control. Values are presented as means ± s.e.m (***P < 0.0001; one-way ANOVA. ns, not significant). C, cartilage; M, meniscus.

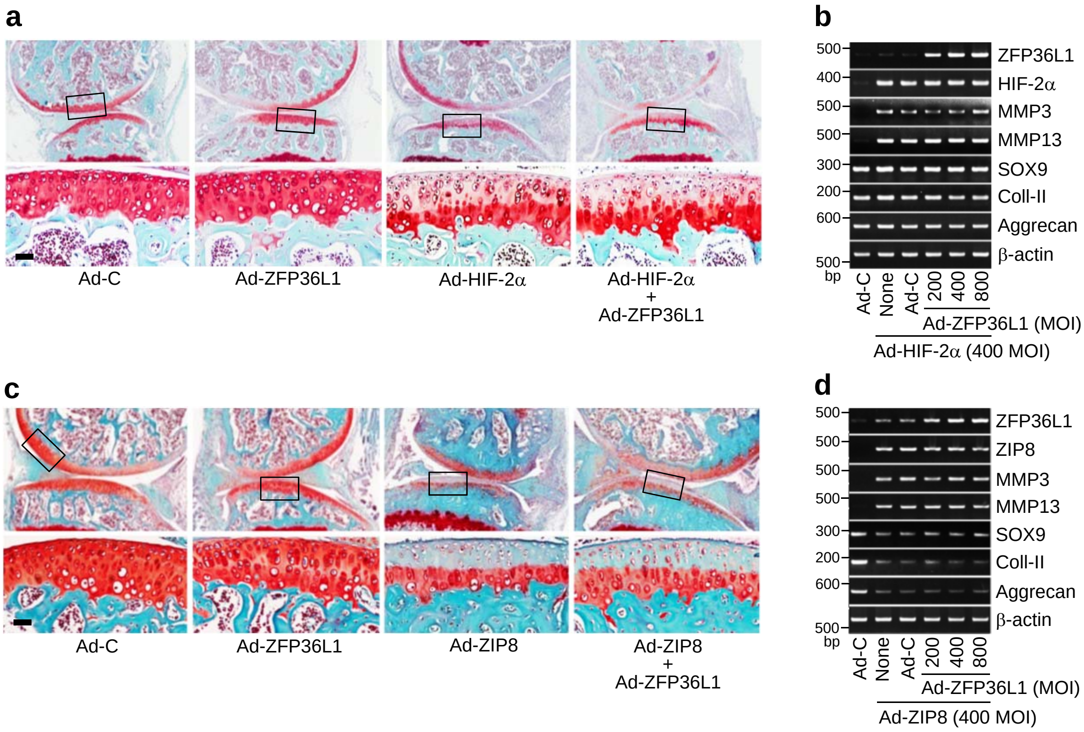

Supplementary Fig. 3. Overexpression of ZFP36L1 does not modulate the OA cartilage destruction caused by overexpression of HIF-2α or ZIP8. (**a, c**) Mice were IA injected with Ad-C as a control, with Ad-HIF-2α to overexpress HIF-2α (**a,** *n* = 12 mice per group), or with Ad-ZIP8 to overexpress ZIP8 (**c,** *n* = 6 mice per group). Ad-ZFP36L1 was co-injected with Ad-HIF-2α or Ad-ZIP8. Cartilage destruction was confirmed by Safranin-O staining at 3 weeks after the IA injections. (**b, d**) RT-PCR analyses of the indicated molecules in chondrocytes infected with Ad-ZFP36L1 with or without Ad-HIF-2α (**b**, *n* = 5) or Ad-ZIP8 (**d**, *n* = 5).

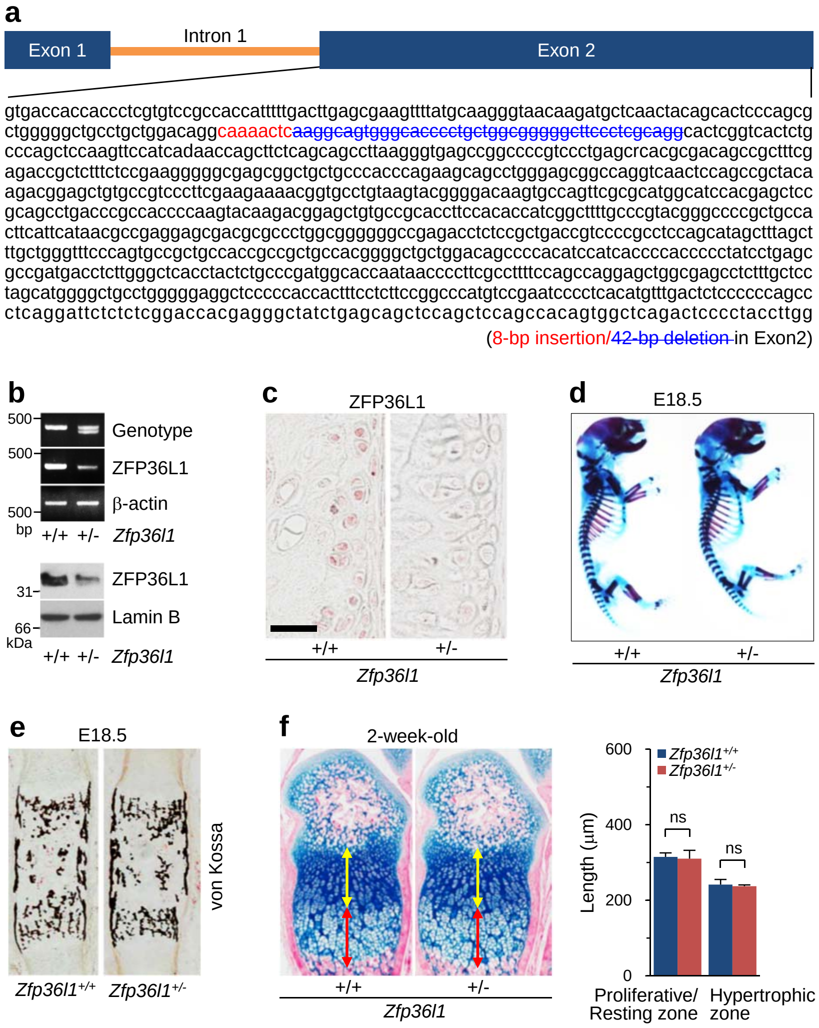

Supplementary Fig. 4. Characterization of *Zfp36l1*$^{+/-}$ mice. (**a**) An 8-bp insertion and a 42-bp deletion in exon2 of *Zfp36l1* were used to generate *Zfp36l1*$^{+/-}$ mice. (**b**) Genotypes and mRNA and protein levels of ZFP36L1 in chondrocytes of *Zfp6l1*$^{+/-}$ mice and their WT littermates, as determined by PCR and Western blotting. β-actin and lamin B were used as loading controls. (**c**) Immunohistochemical staining of ZFP36L1 in the cartilage sections of 10-week-old *Zfp36l1*$^{+/-}$ mice and their WT littermates. (**d-f**) Skeletal staining of E18.5 embryos (**d**), von Kossa staining of E18.5 metatarsal bone (**e**), Alcian blue/nuclear fast red staining of metatarsal bones from 2-week-old mice, and the lengths of the indicated zones (**f**) in *Zfp36l1*$^{+/-}$ mice and WT littermates (n = 7 mice per group). Values are presented as means ± s.e.m.; ns, not significant by two-tailed t-test. Scale bar: 50 μm.

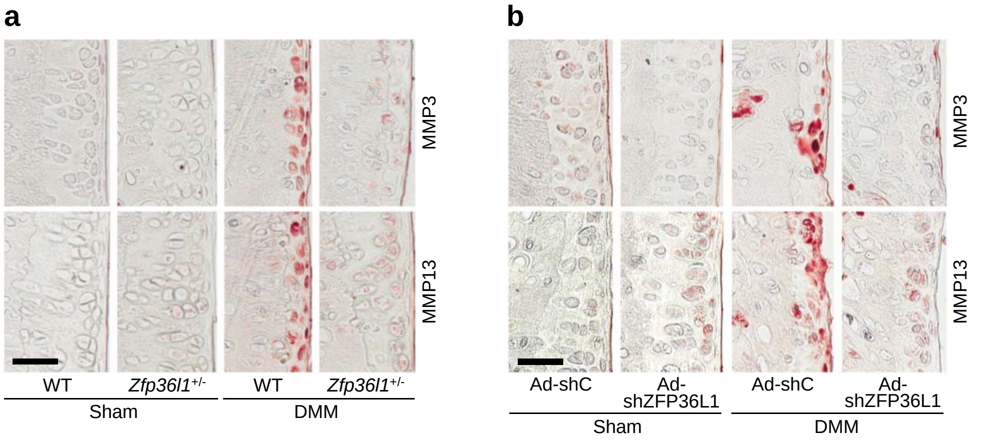

Supplementary Fig. 5. Expression of MMP3 and MMP13 in DMM-operated cartilage tissue. (**a, b**) Representative immunostaining images of MMP3 and MMP13 in cartilage sections of sham- or DMM-operated WT and *Zfp36l1*$^{+/-}$ mice (**a**, n = 5 mice per group) and WT mice subjected to IA injection with Ad-shC or Ad-shZFP36L1 (**b**, n = 5 mice per group). Scale bar: 50 μm.

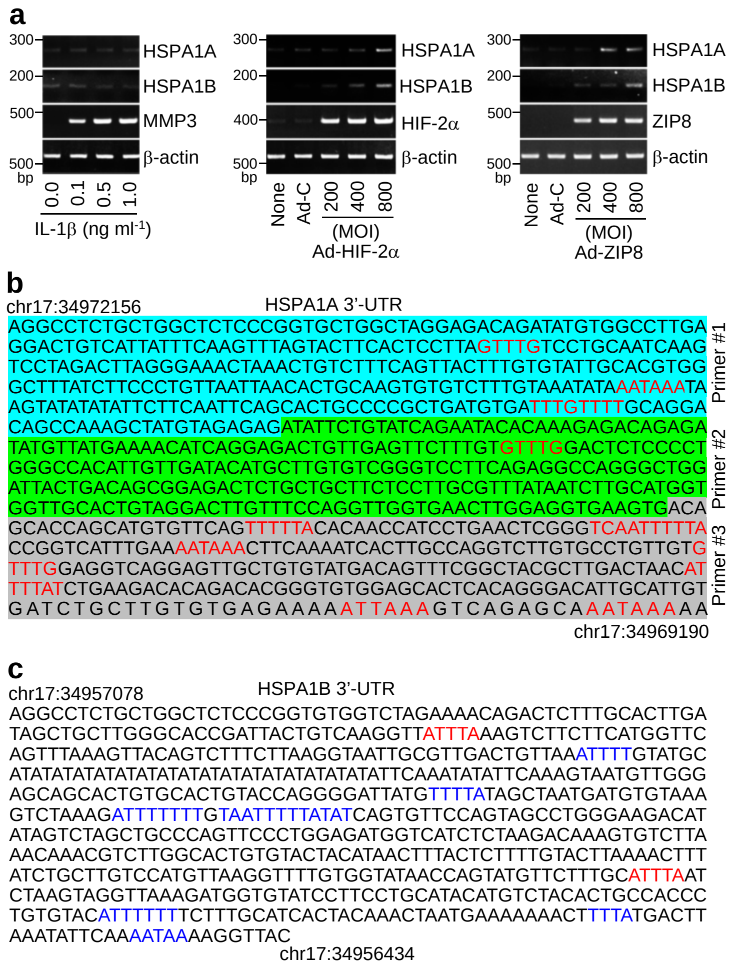

Supplementary Fig. 6. The mRNA of HSPA1A and HSPA1B are targets of ZFP36L1. (**a**) Representative RT-PCR results for the indicated mRNAs in chondrocytes treated with IL-1β or infected with 800 MOI of control virus (Ad-C) or the indicated MOIs of Ad-HIF-2α or Ad-ZIP8 (n = 5). (**b**) Sequence analysis of the AREs in the 3'-UTR of the HSPA1A mRNA. (**c**) Sequence analysis of the AREs in the 3'-UTR of the HSPA1B mRNA.

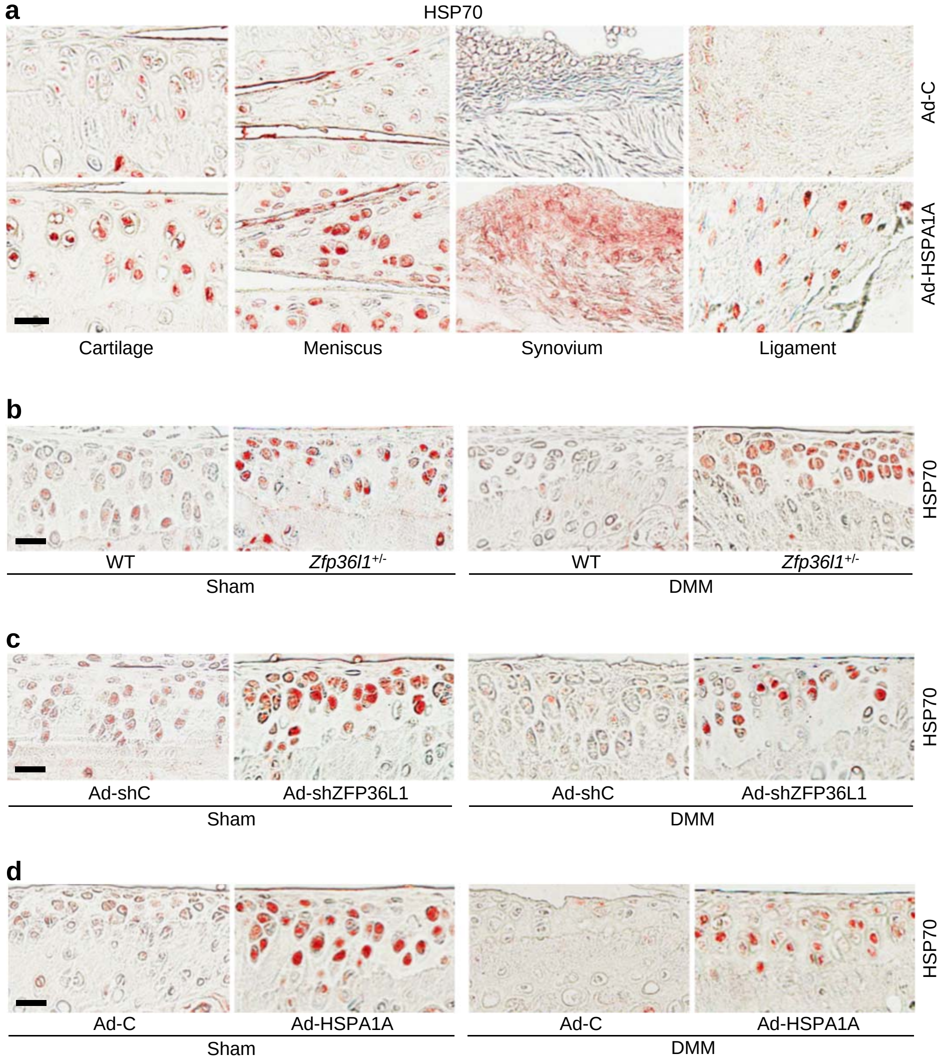

Supplementary Fig. 7. Expression of HSP70 in cartilage tissue. (**a**) Representative immunostaining images of HSP70 in the indicated joint sections of mice IA injected with Ad-C or Ad-HSPA1A (*n* = 6 mice per group). (**b**) Representative immunostaining images of HSP70 in cartilage of sham- or DMM-operated *Zfp36l1*$^{+/-}$ and WT mice (*n* = 6 mice per group). (**c**) Representative immunostaining images of HSP70 in cartilage of sham- or DMM-operated mice IA injected with Ad-shC or Ad-shZFP36L1 (*n* = 6 mice per group). (**d**) Representative immunostaining images of HSP70 in cartilage of sham- or DMM-operated mice IA injected with Ad-C or Ad-HSPA1A (*n* = 6 mice per group).

a

mRNA	Class of ARE (AUUUA)	Number
MMP3	IIE	1
MMP13	IIE	5
ADAMTS5	IIE	15
	I	1
SOX9	I	6
COL2A1	IID	1
ACAN	IIE	1

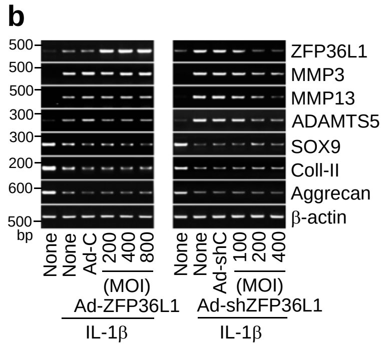

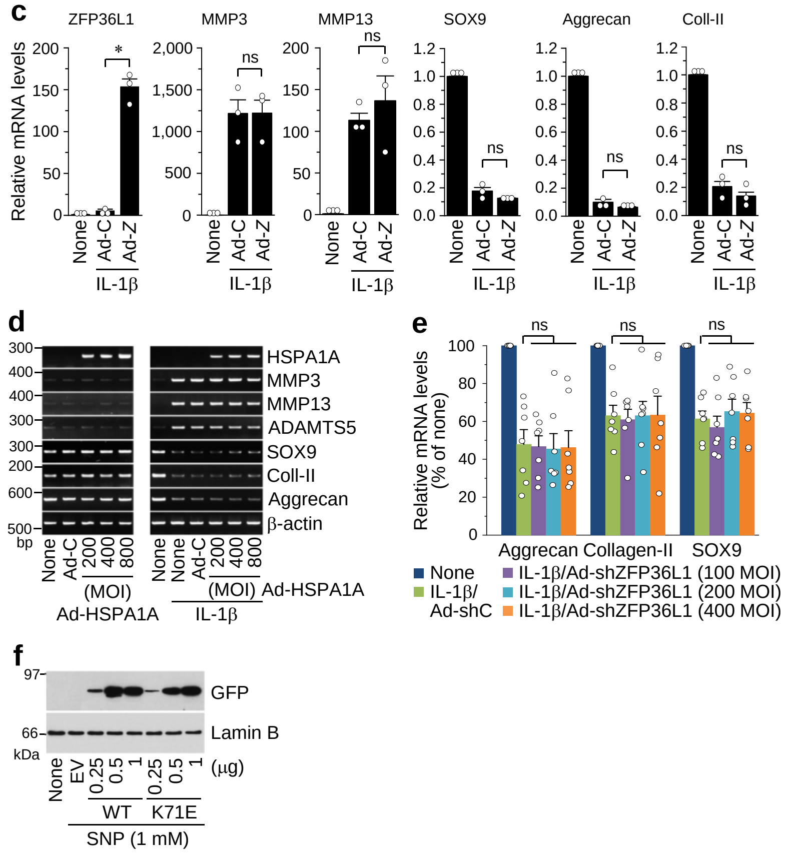

Supplementary Fig. 8. Matrix-degrading enzymes and cartilage ECM molecules are not direct targets of ZFP36L1 in chondrocytes. (**a**) The AREs in the 3'-UTRs of the indicated molecules. (**b**) RT-PCR analysis of the indicated molecules in chondrocytes infected with Ad-ZFP36L1 or Ad-shZFP36L1 in the absence or presence of IL-1β (1 ng ml^{-1} for 24 hours). (**c**) qRT-PCR analysis (n = 3) of the indicated molecules in chondrocytes infected with Ad-C or Ad-ZFP36L1 (Ad-Z) followed by exposure to IL-1β (1 ng ml^{-1} for 24 hours). (**d**) RT-PCR analysis of the indicated molecules in chondrocytes infected with Ad-HSPA1A with or without IL-1β treatment (1 ng ml^{-1} for 24 h). (**e**) qRT-PCR analysis of the indicated molecules in chondrocytes infected with Ad-shC as a control or with Ad-shZFP36L1 (n = 7), followed by exposure to IL-1β (1 ng ml^{-1} for 24 hours). (**f**) Chondrocytes were transfected with vectors encoding WT-HSPA1A or K71E-HSPA1A in the presence or absence of SNP (1 mM) for 12 hours. Representative Western blot images of flagged GFP (n = 4). Values are presented as means ± s.e.m. (*P < 0.01; ns, not significant; two-tailed t-test (c) and one-way ANOVA (e). β-actin and lamin B were used as a loading control.

Supplementary Fig. 9. Uncropped scans of the gel images. (**a-l**) Uncropped immunoblots related to Figures 2c (**a**), 4c (**b**), 4d (**c**), 4e (**d**), S1b (**e**), S1a (**f**), S3 (**g**), S4b (**h**), S6a (**i**), S8b (**j**), S8d (**k**), and S8f (**l**).

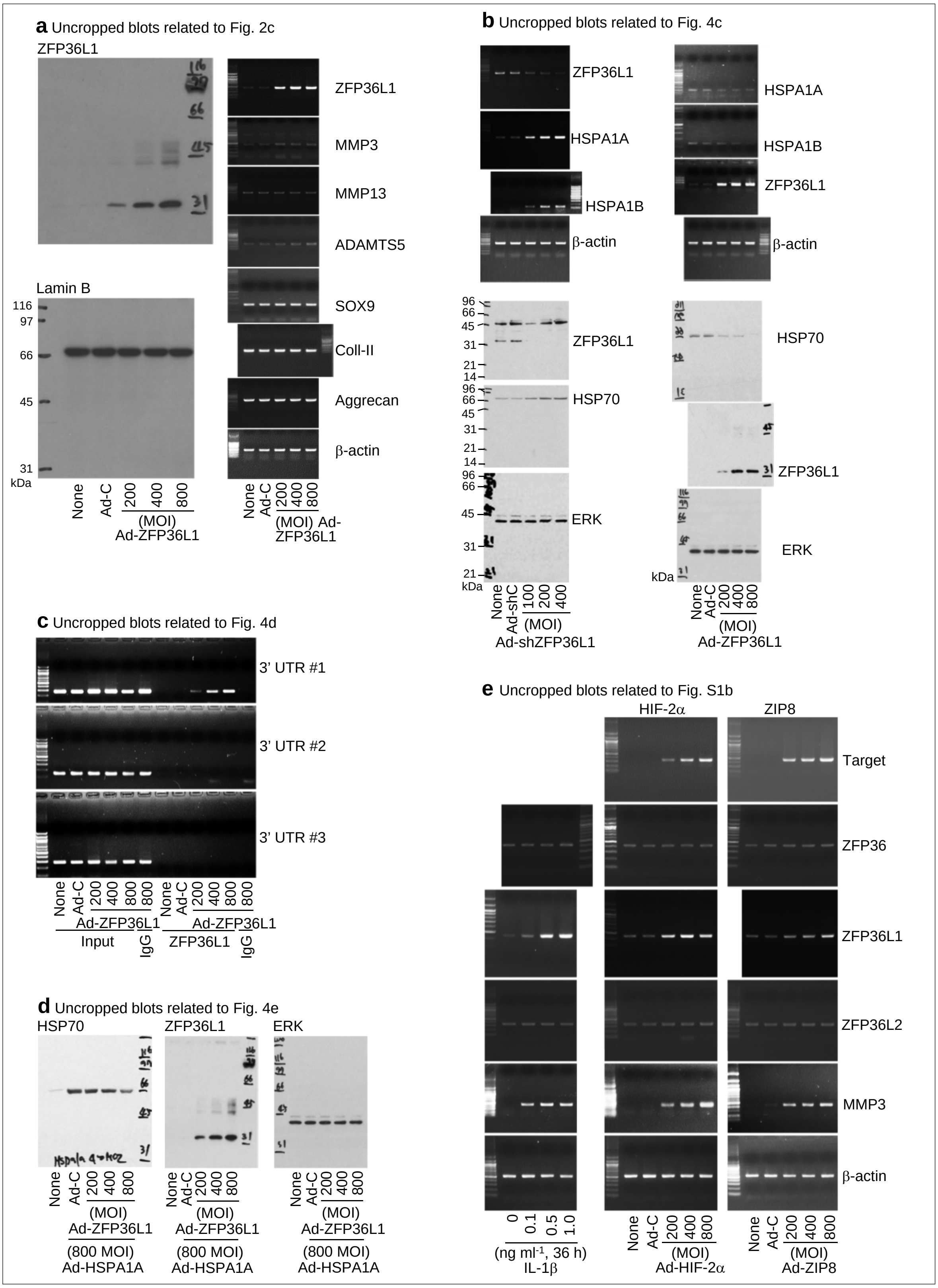

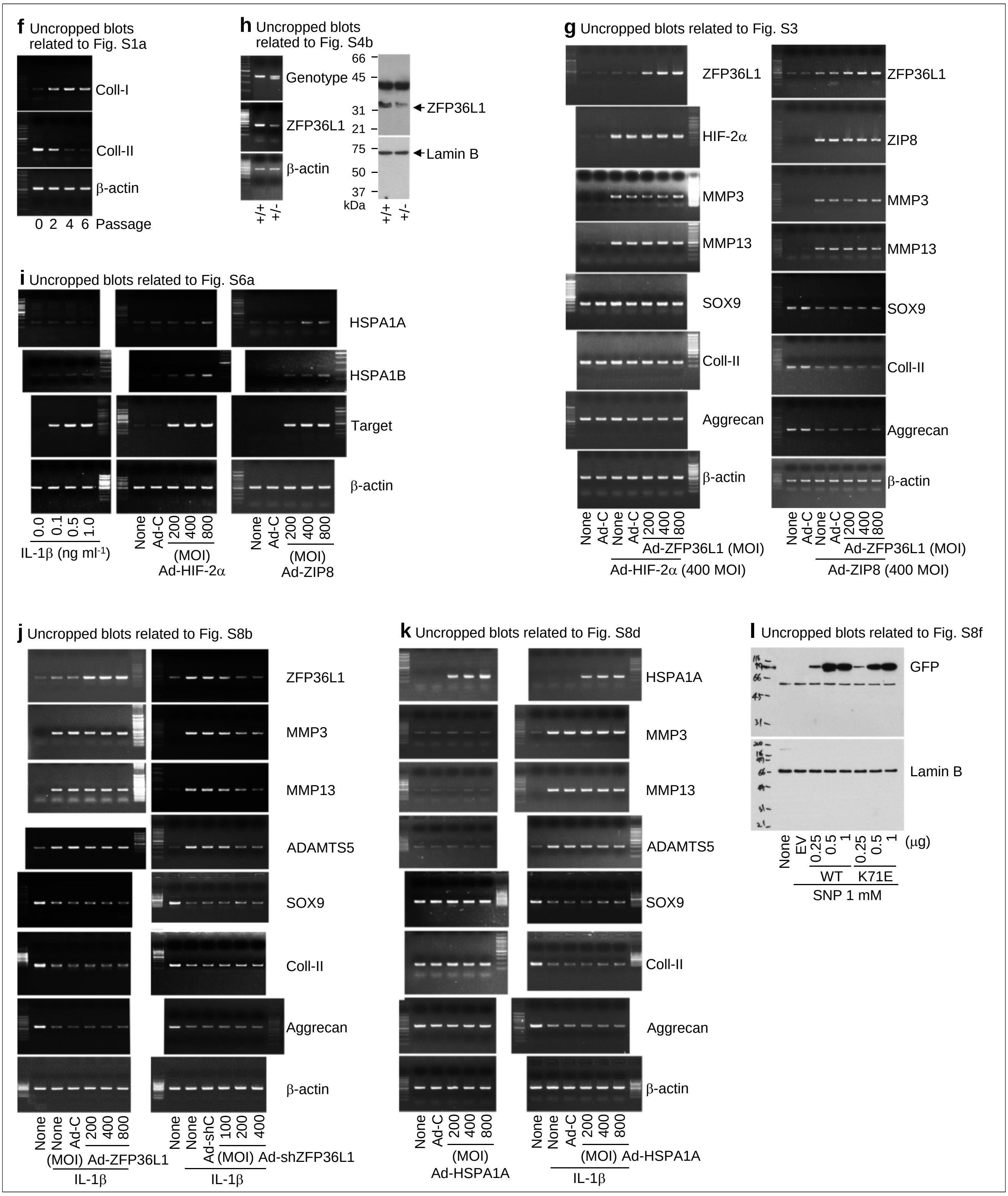
f Uncropped blots related to Fig. S1a
Coll-I
Coll-II
β-actin
0 2 4 6 Passage
h Uncropped blots related to Fig. S4b
Genotype
ZFP36L1
β-actin
+/+ +/-
66
45
31
21
75
50
37
kDa
ZFP36L1
Lamin B
+/+ +/-
g Uncropped blots related to Fig. S3
ZFP36L1
HIF-2α
MMP3
MMP13
SOX9
Coll-II
Aggrecan
β-actin
None Ad-C None Ad-C 200 400 800
Ad-ZFP36L1 (MOI)
Ad-HIF-2α (400 MOI)
ZFP36L1
ZIP8
MMP3
MMP13
SOX9
Coll-II
Aggrecan
β-actin
None Ad-C None Ad-C 200 400 800
Ad-ZFP36L1 (MOI)
Ad-ZIP8 (400 MOI)
i Uncropped blots related to Fig. S6a
HSPA1A
HSPA1B
Target
β-actin
0.0 0.1 0.5 1.0
IL-1β (ng ml-1)
None Ad-C 200 400 800
(MOI)
Ad-HIF-2α
None Ad-C 200 400 800
(MOI)
Ad-ZIP8
j Uncropped blots related to Fig. S8b
ZFP36L1
MMP3
MMP13
ADAMTS5
SOX9
Coll-II
Aggrecan
β-actin
None None Ad-C 200 400 800
(MOI) Ad-ZFP36L1
IL-1β
None None Ad-shC 100 200 400
(MOI) Ad-shZFP36L1
IL-1β
k Uncropped blots related to Fig. S8d
HSPA1A
MMP3
MMP13
ADAMTS5
SOX9
Coll-II
Aggrecan
β-actin
None Ad-C 200 400 800
(MOI)
Ad-HSPA1A
None None Ad-C 200 400 800
(MOI) Ad-HSPA1A
IL-1β
l Uncropped blots related to Fig. S8f
GFP
Lamin B
None EV 0.25 0.5 1 0.25 0.5 1 (μg)
WT K71E
SNP 1 mM

Supplementary Table 1. mRNA levels of 114 genes involved in mRNA catabolic process in chondrocytes treated with IL-1β (1 ng ml^{-1}, 36 h) or infected with 800 MOI of Ad-HIF-2α or Ad-ZIP8 (36 h).

Gene symbol	Definition	Fold change		
		IL-1β	Ad-HIF-2α	Ad-ZIP8
Ago1	Argonaute 1	1.14 ± 0.14	1.09 ± 0.08	1.10 ± 0.08
Ago2	Argonaute 2	1.07 ± 0.05	1.15 ± 0.10	1.04 ± 0.08
Ago3	Argonaute3	0.86 ± 0.07	1.10 ± 0.15	1.03 ± 0.13
Ago4	Argonaute 4	1.27 ± 0.15	0.94 ± 0.17	0.91 ± 0.14
Atm	Serine-protein kinase ATM	0.78 ± 0.09	1.05 ± 0.16	1.19 ± 0.14
Auh	ethylglutaconyl-CoA hydratase	1.56 ± 0.23	1.27 ± 0.24	1.19 ± 0.22
Casc3	Protein CASC3	1.00 ± 0.04	0.89 ± 0.03	0.93 ± 0.04
Cnot1	CCR4-NOT transcription complex subunit 1	1.14 ± 0.04	1.04 ± 0.11	1.00 ± 0.09
Cnot2	CCR4-NOT transcription complex subunit 2	1.03 ± 0.12	0.94 ± 0.04	0.96 ± 0.06
Cnot3	CCR4-NOT transcription complex subunit 3	0.92 ± 0.07	1.02 ± 0.07	1.01 ± 0.09
Cnot4	CCR4-NOT transcription complex subunit 4	0.97 ± 0.04	0.97 ± 0.06	0.97 ± 0.07
Cnot6	CCR4-NOT transcription complex subunit 6	0.97 ± 0.14	0.97 ± 0.05	0.96 ± 0.03
Cnot6l	CCR4-NOT transcription complex subunit 6-like	1.01 ± 0.07	0.98 ± 0.10	0.94 ± 0.04
Cnot7	CCR4-NOT transcription complex subunit 7	1.28 ± 0.13	0.92 ± 0.04	0.89 ± 0.04
Cnot8	CCR4-NOT transcription complex subunit 8	0.89 ± 0.09	0.82 ± 0.08	0.81 ± 0.07
Cpeb3	Cytoplasmic polyadenylation element-binding protein 3	1.10 ± 0.13	0.84 ± 0.12	0.86 ± 0.09
Csde1	Cold shock domain-containing protein E1	1.12 ± 0.07	1.09 ± 0.06	1.08 ± 0.06
Dcp1a	mRNA-decapping enzyme 1A	0.99 ± 0.10	0.92 ± 0.08	0.91 ± 0.03
Dcp1b	mRNA-decapping enzyme 1B	0.97 ± 0.09	0.94 ± 0.12	0.99 ± 0.14
Dcp2	m7GpppN-mRNA hydrolase	0.83 ± 0.15	0.89 ± 0.07	1.06 ± 0.06
Dhx34	Probable ATP-dependent RNA helicase DHX34	0.83 ± 0.09	0.89 ± 0.07	0.90 ± 0.08
Dis3	Exosome complex exonuclease RRP44	0.58 ± 0.01	0.78 ± 0.07	0.83 ± 0.07
Dis3l	DIS3-like exonuclease 1	0.86 ± 0.05	0.97 ± 0.13	1.00 ± 0.19
Dis3l2	DIS3-like exonuclease 2	1.11 ± 0.11	1.08 ± 0.07	1.05 ± 0.08
Dxo	Decapping and exoribonuclease protein	1.05 ± 0.10	1.01 ± 0.11	0.97 ± 0.15
Etf1	Eukaryotic peptide chain release factor subunit 1	0.82 ± 0.08	0.91 ± 0.04	0.92 ± 0.05
Eif3e	Eukaryotic translation initiation factor 3 subunit E	1.01 ± 0.21	0.97 ± 0.07	1.03 ± 0.09
Eif4a3	Eukaryotic initiation factor 4A-III	0.85 ± 0.14	0.95 ± 0.13	0.96 ± 0.12

Eri1	3-5 exoribonuclease 1	0.71 ± 0.10	1.10 ± 0.10	1.13 ± 0.12
Exosc2	Exosome complex component RRP4	0.65 ± 0.03	0.92 ± 0.10	0.95 ± 0.11
Exosc3	Exosome complex component RRP40	0.94 ± 0.10	1.00 ± 0.13	0.98 ± 0.11
Exosc4	Exosome complex component RRP41	0.81 ± 0.10	0.87 ± 0.07	0.93 ± 0.09
Exosc5	Exosome complex component RRP46	0.70 ± 0.04	0.93 ± 0.07	1.05 ± 0.06
Exosc7	Exosome complex component RRP42	0.87 ± 0.07	0.87 ± 0.11	0.93 ± 0.12
Exosc8	Exosome complex component RRP43	0.56 ± 0.05	1.04 ± 0.21	1.05 ± 0.27
Exosc9	Exosome complex component RRP45	0.86 ± 0.04	0.90 ± 0.07	0.92 ± 0.05
Exosc10	Exosome component 10	1.05 ± 0.06	1.07 ± 0.07	1.13 ± 0.04
Gspt1	Eukaryotic peptide chain release factor GTP-binding subunit ERF3A	0.76 ± 0.09	0.90 ± 0.08	0.92 ± 0.09
Gspt2	Eukaryotic peptide chain release factor GTP-binding subunit ERF3B	1.22 ± 0.10	1.12 ± 0.18	1.08 ± 0.20
Gtpbp1	GTP-binding protein 1	0.88 ± 0.05	1.05 ± 0.11	1.00 ± 0.10
Gtpbp2	GTP-binding protein 2	0.90 ± 0.05	1.09 ± 0.18	1.10 ± 0.20
Hnrnpr	Heterogeneous nuclear ribonucleoprotein R	0.94 ± 0.10	0.99 ± 0.08	1.00 ± 0.11
Khsrp	Far upstream element-binding protein 2	0.95 ± 0.06	1.01 ± 0.07	1.02 ± 0.07
Lsm1	U6 snRNA-associated Sm-like protein LSm1	0.86 ± 0.07	1.02 ± 0.07	0.98 ± 0.04
Lsm2	U6 snRNA-associated Sm-like protein LSm2	0.71 ± 0.13	1.05 ± 0.16	1.04 ± 0.09
Lsm3	U6 snRNA-associated Sm-like protein LSm3	0.73 ± 0.18	1.07 ± 0.19	1.22 ± 0.21
Lsm4	U6 snRNA-associated Sm-like protein LSm4	0.74 ± 0.12	0.94 ± 0.09	1.02 ± 0.13
Lsm5	U6 snRNA-associated Sm-like protein LSm5	0.73 ± 0.13	0.95 ± 0.21	0.92 ± 0.24
Lsm6	U6 snRNA-associated Sm-like protein LSm6	0.93 ± 0.04	1.07 ± 0.16	1.18 ± 0.17
Lsm7	U6 snRNA-associated Sm-like protein LSm7	0.68 ± 0.09	0.83 ± 0.13	0.99 ± 0.16
Magoh	Protein mago nashi homolog	0.75 ± 0.09	0.92 ± 0.05	0.87 ± 0.10
Magohb	Protein mago nashi homolog 2	0.72 ± 0.13	0.82 ± 0.18	0.83 ± 0.16
Mex3d	RNA-binding protein MEX3D	1.16 ± 0.08	0.99 ± 0.16	1.09 ± 0.13
Mir466l	microRNA 466l	1.21 ± 0.12	1.08 ± 0.34	0.78 ± 0.23
Mirlet7b	MicroRNA Let-7b	1.28 ± 0.60	1.07 ± 0.13	1.17 ± 0.17
Mirlet7c-1	MicroRNA Let-7c	1.19 ± 0.48	1.06 ± 0.28	1.06 ± 0.11
Mirlet7c-2	microRNA let7c-2	0.88 ± 0.33	0.97 ± 0.33	1.00 ± 0.30
Mtpap	Poly(A) RNA polymerase, mitochondrial	0.98 ± 0.10	0.89 ± 0.07	0.94 ± 0.07
Nanos1	Nanos homolog 1	0.91 ± 0.17	1.02 ± 0.07	1.02 ± 0.06
Nanos2	Nanos homolog 2	0.85 ± 0.22	0.94 ± 0.09	0.97 ± 0.08

Nanos3	Nanos homolog 3	1.01 ± 0.18	0.99 ± 0.23	1.02 ± 0.24
Nbas	Neuroblastoma-amplified sequence	0.65 ± 0.09	0.77 ± 0.06	0.74 ± 0.03
Ncbp1	Nuclear cap-binding protein subunit 1	0.85 ± 0.09	1.09 ± 0.09	1.05 ± 0.07
Ncbp2	Nuclear cap-binding protein subunit 2	0.91 ± 0.04	0.85 ± 0.08	0.91 ± 0.08
Nudt16	U8 snoRNA-decapping enzyme	1.16 ± 0.09	0.98 ± 0.08	0.94 ± 0.10
Pabpc1	Polyadenylate-binding protein 1	1.09 ± 0.05	1.05 ± 0.03	1.07 ± 0.05
Pan2	PAN2-PAN3 deadenylation complex catalytic subunit PAN2	0.90 ± 0.04	1.03 ± 0.09	0.96 ± 0.13
Pan3	AN2-PAN3 deadenylation complex subunit PAN3	1.13 ± 0.11	1.01 ± 0.04	1.01 ± 0.04
Papd4	Poly(A) RNA polymerase GLD2	0.63 ± 0.15	0.72 ± 0.06	0.75 ± 0.07
Papd5	Non-canonical poly(A) RNA polymerase PAPD5	0.98 ± 0.07	1.00 ± 0.08	1.00 ± 0.08
Parn	Poly(A)-specific ribonuclease PARN	0.86 ± 0.08	0.91 ± 0.06	0.99 ± 0.06
Pelo	Protein pelota homolog	0.91 ± 0.03	0.87 ± 0.06	0.87 ± 0.07
Polr2d	DNA-directed RNA polymerase II subunit RPB4	1.21 ± 0.14	1.03 ± 0.15	0.96 ± 0.14
Polr2g	DNA-directed RNA polymerase II subunit RPB7	0.85 ± 0.06	0.77 ± 0.05	0.87 ± 0.06
Ptbp1	Polypyrimidine tract-binding protein 1	0.81 ± 0.07	1.03 ± 0.03	1.01 ± 0.02
Pnpt1	Polyribonucleotide nucleotidyltransferase 1, mitochondrial	0.79 ± 0.16	0.88 ± 0.07	0.95 ± 0.06
Pnrc2	Proline-rich nuclear receptor coactivator 2	0.84 ± 0.07	0.83 ± 0.10	0.91 ± 0.08
Pym1	Partner of Y14 and mago	0.96 ± 0.10	1.12 ± 0.10	1.10 ± 0.09
Rbm8a	RNA-binding protein 8A	0.93 ± 0.14	0.86 ± 0.12	0.89 ± 0.11
Rbm8a2	RNA binding motif protein 8A2	0.97 ± 0.13	0.99 ± 0.13	1.16 ± 0.21
Rc3h1	Roquin-1	1.43 ± 0.11	1.22 ± 0.13	1.17 ± 0.12
Rnps1	RNA-binding protein with serine-rich domain 1	0.88 ± 0.06	0.93 ± 0.09	0.99 ± 0.08
Rqcd1	CCR4-NOT transcription complex subunit 9	0.73 ± 0.06	0.92 ± 0.06	0.98 ± 0.07
Secisbp2	Selenocysteine insertion sequence-binding protein 2	0.91 ± 0.04	0.90 ± 0.07	0.87 ± 0.08
Smg1	Serine/threonine-protein kinase SMG1	1.12 ± 0.07	0.96 ± 0.17	0.96 ± 0.15
Smg5	Protein SMG5	0.93 ± 0.06	0.89 ± 0.04	0.90 ± 0.06
Smg6	Telomerase-binding protein EST1A	1.02 ± 0.11	0.93 ± 0.06	0.93 ± 0.06
Smg7	Protein SMG7	0.94 ± 0.06	0.86 ± 0.05	0.88 ± 0.04
Smg8	Protein SMG8	0.92 ± 0.17	1.05 ± 0.07	1.06 ± 0.07
Smg9	Protein SMG9	1.20 ± 0.23	1.05 ± 0.05	1.09 ± 0.05
Supv3l1	ATP-dependent RNA helicase SUPV3L1, mitochondrial	0.85 ± 0.07	0.84 ± 0.03	0.87 ± 0.03
Thrap3	Thyroid hormone receptor-associated protein 3	0.93 ± 0.15	0.90 ± 0.07	0.90 ± 0.05
Tnrc6a	Trinucleotide repeat-containing gene 6A protein	1.06 ± 0.12	0.95 ± 0.03	0.96 ± 0.05

Tnrc6b	Trinucleotide repeat-containing gene 6B protein	0.96 ± 0.09	0.99 ± 0.26	0.98 ± 0.15
Tnrc6c	Trinucleotide repeat-containing gene 6C protein	0.95 ± 0.04	0.90 ± 0.04	0.94 ± 0.04
Tob1	Protein Tob1	1.60 ± 0.17	1.11 ± 0.15	1.05 ± 0.14
Tut1	Speckle targeted PIP5K1A-regulated poly(A) polymerase	0.90 ± 0.07	0.87 ± 0.05	0.88 ± 0.06
Uhmk1	Serine/threonine-protein kinase Kist	0.91 ± 0.07	0.88 ± 0.08	0.85 ± 0.07
Upf1	Regulator of nonsense transcripts 1	0.81 ± 0.05	0.94 ± 0.10	0.98 ± 0.08
Upf2	Regulator of nonsense transcripts 2	1.10 ± 0.27	1.00 ± 0.05	0.97 ± 0.06
Upf3b	Regulator of nonsense transcripts 3B	1.05 ± 0.24	1.11 ± 0.05	1.04 ± 0.08
Wdr61	WD repeat-containing protein 61	0.98 ± 0.07	0.89 ± 0.10	0.97 ± 0.10
Xrn1	5-3 exoribonuclease 1	1.21 ± 0.14	1.03 ± 0.14	1.00 ± 0.11
Zc3h12d	Probable ribonuclease ZC3H12D	0.94 ± 0.18	0.86 ± 0.11	0.96 ± 0.19
Zc3hav1	Zinc finger CCCH-type antiviral protein 1	0.81 ± 0.04	1.50 ± 0.16	1.24 ± 0.06
Zcchc6	Terminal uridylyltransferase 7	1.46 ± 0.13	1.02 ± 0.05	0.97 ± 0.04
Zcchc11	Terminal uridylyltransferase 4	1.07 ± 0.10	0.93 ± 0.11	0.94 ± 0.07
Zfp36	mRNA decay activator protein ZFP36	1.05 ± 0.09	1.34 ± 0.29	1.14 ± 0.17
Zfp36l1	mRNA decay activator protein ZFP36L1	3.32 ± 0.39	2.07 ± 0.26	1.86 ± 0.28
Zfp36l2	mRNA decay activator protein ZFP36L2	1.14 ± 0.12	1.49 ± 0.19	1.55 ± 0.20
Zfp36l3	mRNA decay activator protein ZFP36L3	0.88 ± 0.09	1.05 ± 0.10	1.06 ± 0.14
Zhx2	Zinc fingers and homeoboxes protein 2	0.94 ± 0.09	1.01 ± 0.10	1.01 ± 0.07
Zpr1	Zinc finger protein ZPR1	0.64 ± 0.05	0.86 ± 0.07	0.93 ± 0.06

Supplementary Table 2. List of top 50 upregulated genes following knockdown of ZFP36L1 via Ad-shZFP36L1 infection in chondrocytes. Chondrocytes were infected with Ad-shZFP36L1 (400 MOI) or Ad-ZFP36L1 (800 MOI) for 36 h. Fold changes against Ad-shControl or Ad-Control are presented.

Gene symbol	Definition	Accession number	Fold change	
			Ad-shZFP36L1	Ad-ZFP36L1
Hspa1a	Heat shock protein 1A	NM_007564	16.803	1.256
Hspa1b	Heat shock protein 1B	NM_010478	10.970	1.218
Gm20481	Predicted gene 20481	ENSMUST00000173680	2.972	1.081
Nov	Nephroblastoma-overexpressed gene	NM_010930	2.794	1.688
Gm13238	Carnitine deficiency-associated gene expressed in ventricle 3 pseudogene	NR_033612	2.578	0.761
Tnfrsf22	TNF receptor superfamily, member 22	NM_001311145	2.543	1.404
Pof1b	Premature ovarian failure 1B	NM_181579	2.386	1.174
Acsm3	Acyl-CoA synthetase medium-chain family member 3	NM_016870	2.316	0.875
Samd5	Sterile α motif domain-containing 5	NM_177271	2.252	1.537
Tnfrsf23	TNF receptor superfamily, member 23	NM_001308111	2.243	1.683
Igkj1	Immunoglobulin kappa joining 1	OTTMUST00000133379	2.202	0.810
Fgl1	Fibrinogen-like protein 1	NM_145594	2.168	1.047
Snora17	Small nucleolar RNA, H/ACA box 17	NR_028571	2.154	1.138
Rerg	RAS-like, estrogen-regulated growth-inhibitor	NM_001164212	2.145	1.384
Rbm46	RNA-binding motif protein 46	NM_001277170	2.128	0.905
Hspb1	Heat shock protein 1	NM_013560	2.078	1.039
Scel	Sciellin	NM_022886	2.057	1.053
Gm3776	Predicted gene 3776	NM_001243092	2.015	1.384
Stfa2	Stefin A2	NM_001082545	1.999	1.216
Cnksr2	Connector enhancer of kinase suppressor of Ras 2	NM_001310719	1.988	0.998
Mettl7a1	Methyltransferase-like 7A1	NM_027334	1.979	0.816
2810474O19 Rik	RIKEN cDNA 2810474O19 gene	NM_001289661	1.975	1.433
Ppif	Peptidylprolyl isomerase F	NM_134084	1.974	1.117
Rgs2	Regulator of G-protein signaling 2	NM_009061	1.953	1.370

Enpep	Glutamyl aminopeptidase	NM_007934	1.930	0.994
Mir743	microRNA 743	NR_030532	1.927	1.015
Gpnmb	Glycoprotein (transmembrane) nmb	NM_053110	1.883	1.371
Prg4	Proteoglycan 4	NM_001110146	1.868	1.468
Abcb1b	ATP-binding cassette, sub-family B, member 1B	NM_011075	1.867	1.598
Osgin1	Oxidative stress-induced growth inhibitor 1	NM_027950	1.863	0.957
Myd88	Myeloid differentiation primary response gene 88	NM_010851	1.858	1.020
Pde7b	Phosphodiesterase 7B	NM_013875	1.858	0.815
4933400C23 Rik	RIKEN cDNA 4933400C23 gene	NR_040770	1.838	0.980
Sord	Sorbitol dehydrogenase	NM_146126	1.833	1.177
Lrrn3	Leucine-rich repeat protein 3, neuronal	NM_001271708	1.809	1.371
Atp1b2	ATPase, Na+/K+-transporting, beta 2 polypeptide	NM_013415	1.803	1.022
Slitrk6	SLIT and NTRK-like family, member 6	NM_175499	1.800	1.659
5730508B09 Rik	RIKEN cDNA 5730508B09 gene	NM_027482	1.787	1.095
Vmn1r208	Vomeronasal 1 receptor 208	NM_134218	1.780	1.055
Ptgs2	Prostaglandin-endoperoxide synthase 2	NM_011198	1.779	1.479
Gm22740	Predicted gene, 22740	ENSMUST0000 0083439	1.779	1.395
Dsp	Desmoplakin	NM_023842	1.762	1.486
Atp10d	ATPase, class V, type 10D	NM_153389	1.754	1.042
Tle1	Transducin-like enhancer of split 1, homolog of Drosophila E	NM_001285529	1.748	1.070
Robo1	Roundabout homolog 1 (Drosophila)	NM_019413	1.730	1.137
Alox12	Arachidonate 12-lipoxygenase	NM_007440	1.730	1.383
Procr	Protein C receptor, endothelial	NM_011171	1.723	1.146
Cpt1a	Carnitine palmitoyltransferase 1a, liver	NM_013495	1.719	1.221
Tinagl1	Tubulointerstitial nephritis antigen-like 1	NM_001168333	1.717	1.194
Pdgfc	Platelet-derived growth factor, C polypeptide	NM_019971	1.716	1.269

Supplementary Table 3. List of top 50 downregulated genes following overexpression of ZFP36L1 via Ad-ZFP36L1 infection in chondrocytes.

Gene symbol	Definition	Accession number	Fold change	
			Ad-ZFP36L1	Ad-shZFP36L1
Mirlet7f-1	microRNA let7f-1	NR_029731	0.321	0.328
Gm6900	Predicted gene 6900	ENSMUST00000178971	0.501	0.514
Gm23474	Predicted gene, 23474	ENSMUST00000158884	0.513	0.488
Zfp626	Zinc finger protein 626	NM_027703	0.519	0.706
Gm24598	Predicted gene, 24598	ENSMUST00000122510	0.580	0.646
Gm23475	Predicted gene, 23475	ENSMUST00000158879	0.590	0.565
Gm23041	Predicted gene, 23041	ENSMUST00000083868	0.595	1.090
Gm7551	Predicted gene 7551	ENSMUST00000089593	0.607	0.563
Gm24564	Predicted gene, 24564	ENSMUST00000082518	0.607	0.632
Saa3	Serum amyloid A 3	NM_011315	0.615	0.507
Gm22205	Predicted gene, 22205	ENSMUST00000122655	0.625	0.658
Mir669p-1	microRNA 669p-1	NR_037257	0.626	0.505
Mir669p-1	microRNA 669p-1	NR_037257	0.626	0.505
Gm22685	Predicted gene, 22685	ENSMUST00000082848	0.626	0.516
Sorbs2	Sorbin and SH3 domain-containing 2	ENSMUST00000136119	0.633	0.611
Mir493	microRNA 493	NR_030573	0.638	0.916
Gm22455	Predicted gene, 22455	ENSMUST00000082580	0.645	0.609
Mir453	microRNA 453	NR_030559	0.651	0.930
Gm22962	Predicted gene, 22962	ENSMUST00000157962	0.652	0.713
Mir1898	microRNA 1898	NR_035443	0.672	0.635
Gm24655	Predicted gene, 24655	ENSMUST00000178790	0.679	0.509
n-R5s127	Nuclear-encoded rRNA 5S 127	ENSMUST00000179185	0.688	0.660
Gm10132	Predicted gene	ENSMUST00000099431	0.692	0.575
Gm23168	Predicted gene, 23168	ENSMUST00000157156	0.693	0.596

Mir199b	microRNA 199b	NR_029811	0.694	0.487
Rps13-ps1	Ribosomal protein S13, pseudogene 1	OTTMUST00000117085	0.695	0.612
Cdkl5	Cyclin-dependent kinase-like 5	NM_001024624	0.699	0.496
Gm24988	Predicted gene, 24988	ENSMUST00000104460	0.699	0.625
Mir684-2	microRNA 684-2	NR_030455	0.699	0.394
Gm15199	Predicted gene 15199	XR_380275	0.703	0.588
Gm5116	Predicted gene 5116	ENSMUST00000187732	0.704	0.658
Rpl15-ps2	Ribosomal protein L15, pseudogene 2	OTTMUST00000117687	0.713	0.666
Gm10130	Predicted gene 10130	ENSMUST00000133235	0.713	0.422
Gm5801	Ubiquitin-conjugating enzyme E2, J2 homolog pseudogene	NR_002889	0.715	0.636
Clmn	Calmin	NM_001040682	0.721	0.479
Pygo1	Pygopus 1	NM_028116	0.721	0.562
Mrps36-ps1	Mitochondrial ribosomal protein S36, pseudogene 1	OTTMUST00000120473	0.733	0.582
Gm23350	Predicted gene, 23350	ENSMUST00000158429	0.735	0.596
Gm10717	Predicted gene 10717	ENSMUST00000075573	0.736	0.663
AW549877	Expressed sequence AW549877	NM_145930	0.738	0.508
Clcn5	Chloride channel 5	NM_001243762	0.742	0.450
Osgin2	Oxidative stress-induced growth inhibitor family member 2	NM_145950	0.749	0.478
Mir684-1	microRNA 684-1	NR_030454	0.751	0.423
1700054O19 Rik	RIKEN cDNA 1700054O19 gene	XR_383299	0.751	0.641
Gm10268	Predicted gene 10268	ENSMUST00000085340	0.752	0.664
Rps3a2	Ribosomal protein S3A2	OTTMUST00000125706	0.754	0.620
Gm12003	Predicted gene 12003	ENSMUST00000118518	0.760	0.541
Gm13238	Carnitine deficiency-associated gene expressed in ventricle 3 pseudogene	NR_033612	0.761	2.578
Lyst	Lysosomal trafficking regulator	NM_010748	0.762	0.616
Gm5327	Predicted pseudogene 5327	ENSMUST00000182864	0.765	0.643

Supplementary Table 4. PCR primers and conditions.

Genes	Strand	Primer sequence	Size (bp)	Purpose	Origin
Acan	S<br>AS	5′-CTGTCTTTGTCACCCACACATG-3′<br>5′-GAAGACGACATCACCATCCCAG-3′	581	RT-PCR	Mouse
Actb	S<br>AS	5′-ATATCGCTGCGCTGGTCGTC-3′<br>5′-AGGATGGCGTGAGGGAGAGC-3′	517	RT-PCR	Mouse
Adamts5	S<br>AS	5′-GCCATTGTAATAACCCTGCACC-3′<br>5′-TCAGTCCCATCCGTAACCTTTG-3′	292	RT-PCR	Mouse
Col1a1	S<br>AS	5′-TACTACCGGGCCGATGATGCTAAC-3′<br>5′-CAGTGATAGGTGATGTTCTGGGAGGC-3′	467	RT-PCR	Mouse
Col2a1	S<br>AS	5′-CACACTGGTAAGTGGGGCAAGA-3′<br>5′-GGATTGTGTTGTTTCAGGGTTCG-3′	173	RT-PCR	Mouse
Epas1	S<br>AS	5′-CGAGAAGAACGACGTGGTGTTC-3′<br>5′-GTGAAGGCTGGCAGGCTCC-3′	333	RT-PCR	Mouse
Gapdh	S<br>AS	5′-TCACTGCCACCCAGAAGAC-3′<br>5′-TGTAGGCCATGAGGTCCAC-3′	450	RT-PCR	Mouse
Hspa1a	S<br>AS	5′-CAGCGAGGCTGACAAGAAGA-3′<br>5′-CTCCCTTGGGCGCCTG-3′	212	RT-PCR	Mouse
Hspa1b	S<br>AS	5′-ACTGTGCACTGTACCAGGGG-3′<br>5′-AGGGAACTGGGCGGCAGCTAGAC-3′	128	RT-PCR	Mouse
Hspa1a<br>3′ UTR #1	S<br>AS	5′-GGTGCTGGCTAGGAGACAGA-3′<br>5′-GGCTGTCCTGCAAAACAAAT-3′	263	RT-PCR<br>RIP assay	Mouse
Hspa1a<br>3′ UTR #2	S<br>AS	5′-TGGGCCACATTGTTGATACA-3′<br>5′-CACCTCCAAGTTCACCAACC-3′	154	RT-PCR<br>RIP assay	Mouse
Hspa1a<br>3′ UTR #3	S<br>AS	5′-CATGGTGGTTGCACTGTAGG-3′<br>5′-ACAATGCAATGTCCCTGTGA-3′	277	RT-PCR<br>RIP assay	Mouse
Mmp3	S<br>AS	5′-AGG GAT GATGATGCTGGTATGG-3′<br>5′-CCATGTTCTCCAACTGCAAAGG-3′	434	RT-PCR	Mouse
Mmp13	S<br>AS	5′-TGATGGACCTTCTGGTCTTCTGG-3′<br>5′-CATCCACATGGTTGGGAAGTTCT-3′	473	RT-PCR	Mouse
Slc39a8	S<br>AS	5′-GAACAATTGCCTGGATGATCACGC-3′<br>5′-AAGCCGGTTAACATCCCTGCATTC-3′	430	RT-PCR	Mouse
Sox9	S<br>AS	5′-ATGCTATCTTCAAGGCGCTG-3′<br>5′-GACGTCGAAGGTCTCAATGT-3′	272	RT-PCR	Mouse
Zfp36	S<br>AS	5′-CCAAGTGCCAGTTTGCTCAC-3′<br>5′-TGGGGGTAGTAGACCTTCGG-3′	414	RT-PCR	Mouse
Zfp36l1 (1)	S<br>AS	5′-CACCCCAAGTACAAGACGGA-3′<br>5′-GCTAGGAGCAAAGAGGCTCG-3′	348	RT-PCR	Mouse

Zfp36l1 (2)	S<br>AS	5´-CCAGGTCAACTCCAGCCGCTACAGG-3´<br>5´-GGAAAAGGCGAAGGGGTTATTGGTG-3´	439	RT-PCR	Mouse
Zfp36l2	S<br>AS	5´-GTCCAAGACGGAGAAATCCC-3´<br>5´-GGTCCCGAAATTTGCTCTCC-3´	326	RT-PCR	Mouse
Zfp36l1	S<br>AS	5´-AGGTGCTGTCCTTTGTGGAC-3´<br>5´-GGCACTTGTCCCCGTACTTA-3´	435	Genotype	Mouse

S, sense; AS, antisense.
