# Supplementary material for: RNA-binding protein ZFP36L1 regulates osteoarthritis by modulating members of the heat shock protein 70 family
Source: Nat Commun. 2019 Jan 8;10:77. doi: 10.1038/s41467-018-08035-7 (PMC6325149; doi:10.1038/s41467-018-08035-7)
Supplement: Supplementary file 3 — Reporting Summary [file 41467_2018_8035_MOESM3_ESM.pdf]

natureresearch

Corresponding author(s): Jang-Soo Chun

Reporting Summary

Nature Research wishes to improve the reproducibility of the work that we publish. This form provides structure for consistency and transparency in reporting. For further information on Nature Research policies, see Authors & Referees and the Editorial Policy Checklist.

Statistical parameters

When statistical analyses are reported, confirm that the following items are present in the relevant location (e.g. figure legend, table legend, main text, or Methods section).

n/a	Confirmed	
☐	☒	The exact sample size (*n*) for each experimental group/condition, given as a discrete number and unit of measurement
☐	☒	An indication of whether measurements were taken from distinct samples or whether the same sample was measured repeatedly
☐	☒	The statistical test(s) used AND whether they are one- or two-sided<br>*Only common tests should be described solely by name; describe more complex techniques in the Methods section.*
☐	☒	A description of all covariates tested
☐	☒	A description of any assumptions or corrections, such as tests of normality and adjustment for multiple comparisons
☐	☒	A full description of the statistics including central tendency (e.g. means) or other basic estimates (e.g. regression coefficient) AND variation (e.g. standard deviation) or associated estimates of uncertainty (e.g. confidence intervals)
☐	☒	For null hypothesis testing, the test statistic (e.g. *F*, *t*, *r*) with confidence intervals, effect sizes, degrees of freedom and *P* value noted<br>*Give P values as exact values whenever suitable.*
☒	☐	For Bayesian analysis, information on the choice of priors and Markov chain Monte Carlo settings
☒	☐	For hierarchical and complex designs, identification of the appropriate level for tests and full reporting of outcomes
☒	☐	Estimates of effect sizes (e.g. Cohen's *d*, Pearson's *r*), indicating how they were calculated
☐	☒	Clearly defined error bars<br>*State explicitly what error bars represent (e.g. SD, SE, CI)*

Our web collection on statistics for biologists may be useful.

Software and code

Policy information about availability of computer code

Data collection	1) qRT-PCR analysis: StepOne™ Software, version. 2.3<br>2) Statistical analysis: SPSS statistics, Release. 23.0.0.0<br>3) Microarray data analysis: R, version.3.3.2; BioConductor, version. 3.4; Affy (R package)-RMA, version. 1.52.0; Python from Python Software Foundation, version. 3.4.3; StatsModels (Python module)-Statistics, version. 0.8.0
Data analysis	CFX Connect Real-Time PCR Detection System (Bio-Rad) and StepOne software ver 2.3 was used for data analyses of quantitative PCR. All statistical calculations in the paper were performed using the SPSS statistics, Release. 23.0.0.0. Affy (R package)-RMA, version. 1.52.0; Python from Python SoftwareFoundation, version. 3.4.3; StatsModels (Python module)-Statistics, version. 0.8.0 were used for microarray data analysis. OriginPro 2018 (64 bit) SR1 b9.5.1.195 was used as a graphing software.

For manuscripts utilizing custom algorithms or software that are central to the research but not yet described in published literature, software must be made available to editors/reviewers upon request. We strongly encourage code deposition in a community repository (e.g. GitHub). See the Nature Research guidelines for submitting code & software for further information.

Data

Policy information about availability of data

All manuscripts must include a data availability statement. This statement should provide the following information, where applicable:

- Accession codes, unique identifiers, or web links for publicly available datasets
- A list of figures that have associated raw data
- A description of any restrictions on data availability

The microarray data have been deposited to the Gene Expression Omnibus under accession codes GSE104794 (for HIF-2α), GSE104795 (for ZIP8), GSE104793 (for IL-1β), and GSE110581 (for ZFP36L1). All other data supporting the findings of this study are available within the paper and its supplementary information files

Field-specific reporting

Please select the best fit for your research. If you are not sure, read the appropriate sections before making your selection.

☒ Life sciences ☐ Behavioural & social sciences ☐ Ecological, evolutionary & environmental sciences

For a reference copy of the document with all sections, see nature.com/authors/policies/ReportingSummary-flat.pdf

Life sciences study design

All studies must disclose on these points even when the disclosure is negative.

Sample size	Although no statistical methods were used to predetermined sample size in vitro and in vivo analyses, we conducted preliminary experiments to estimate variances in each assay and determined sufficient sample size.
Data exclusions	No samples or animals were excluded from analyses.
Replication	All experimental findings were reproduced independently at least four times. For each figure panel, the numbers of biologically independent samples, mice per group, or human specimens are indicated in the figure legends. Data shown in figure panels are the mean of all independent biological repeats. Western blot pictures, confocal microscopy images, or histological images typically are from a representative experiment; however, the number of independent repeats is clearly indicated in the figure legends and all attempts at replication were successful.
Randomization	For in vitro experiments, cultures were randomly chosen for different treatments and experiments were performed multiple times. For mouse experiments, mutant mice (ZFP36L1+/-) and WT littermates were allocated into groups based on sex, age, and genotype. Male mice were selected for the experiment in order to avoid concerns about hormonal effect in female mice. After the selection, mice for the DMM surgery or intra-articular injection were allocated randomly without subjective judgment.
Blinding	Cartilage destruction analysis, synovial inflammation scoring, osteophyte maturity analysis, SBP thickness analysis, and immunohistochemistry were performed by individuals (Young-Ok Son, Hyo-Eun Kim, Wan-Su Choi, Jiye Yang, Ji-Sun Kwak, Seul Ki Kim) who were blinded to the nature of the mice under analysis (both what specific mouse strains or treatment groups were in the experiment and whether any individual mouse belonged to control versus experimental groups).

Reporting for specific materials, systems and methods

Materials & experimental systems

n/a	Involved in the study	
☒	☐	Unique biological materials
☐	☒	Antibodies
☒	☐	Eukaryotic cell lines
☒	☐	Palaeontology
☐	☒	Animals and other organisms
☐	☒	Human research participants

Methods

n/a	Involved in the study	
☒	☐	ChIP-seq
☒	☐	Flow cytometry
☒	☐	MRI-based neuroimaging

Antibodies

Antibodies used	Immunohistochemistry<br>ZFP36L1 (1:100; Abcam; ab209419)

HSP70 (1:1,000; R&D Systems; AF1663 and 3.5ug ml-1; Abcam; ab5442
MMP3 (4 ug ml-1; Abcam; ab52915)
MMP13 (1:100; Abcam; ab51072)
Western blotting
ZFP36L1 (10 ug ml-1; Santa Cruz Biotechnology; SC-134091)
HSP70 (1:1,000; Cell Signaling Technology; #4872)
GFP (1:4,000; Abcam; ab290)
LaminB (1:2,000; Santa Cruze; sc6216)
ERK (1:2,000; BD Biosciences; 610408)

RIP assay
ZFP36L1 (2ug, FabGennix International, 101AP)
IgG (1ug, EMD Millipore Corp, CS200621)

Validation

All antibodies used in this study were validated by the suppliers as follows:
ZFP36L1 (1:100; Abcam; ab209419) for IHC and WB: species (Human, mouse, Rat), application (IHC), manufacturer's website (https://www.abcam.com/tis11b-antibody-ab209419.html)
ZFP36L1 (10 ug ml-1; Santa Cruz Biotechnology; SC-134091) for WB: species (Human, mouse, Rat), application (WB, IHC, IP,ELISA), manufacturer's website (http://datasheets.scbt.com/sc-134091.pdf)
ZFP36L1 (2ug; FabGennix International; 101AP) for RIP assay: species (Human, Monkey, mouse, Rat), application (ELISA, WB, IHC, IP), manufacturer's website (https://fabgennix.com/ZFP36L1-Antibody)
IgG (1ug: EMD Milipore Corp; CS200621) for RIP assay: species (mouse), application (Normal Mouse IgG Polyclonal Antibody control validated for use in Immunoprecipitation & Western Blotting), manufacturer's website (http://www.merckmillipore.com/KR/ko/product/Normal-Mouse-IgG,MM_NF-12-371?ReferrerURL=https%3A%2F%2Fwww.google.co.kr%2F)
HSP70 (1:1,000; R&D Systems; AF1663) for IHC: species (Human, mouse, Rat), application (WB, IHC), manufacturer's website (https://www.rndsystems.com/products/human-mouse-rat-hsp70-hspa1a-antibody_af1663)
HSP70 (3.5 ug ml-1; Abcam; ab5442) for IHC and WB: species (Human, mouse, Rat, Chicken), application (Flow cyt, WB, IHC-P, IP, IF), manufacturer's website (https://www.abcam.com/hsp70-antibody-2a4-ab5442.html)
HSP70 (1:1,000; Cell Signaling Technology; #4872) for IHC and WB: species (Human, mouse, Rat, Monkey, Bovine), application (WB, IHC, IP, IF, CHIP), manufacturer's website (https://www.cellsignal.com/products/primary-antibodies/hsp70-antibody/4872)
MMP3 (1: 100; Abcam; ab52915; Lot#GR1299549) for IHC: species (Human, Mouse, Rat), application (WB, IHC-P, IF, ICC), manufacturer's website (https://www.abcam.com/mmp3-antibody-ep1186y-ab52915.html)
MMP13 (1:100; Abcam; ab39012; Lot#GR149380-1) for IHC: species (Human, Mouse, Rat, Dog), application (WB, IHC-P, IF, ELISA, ICC, Flow Cyt), manufacturer's website (https://www.abcam.com/mmp13-antibody-ab39012.htm)
GFP(1:4,000;Abcam; ab290) for WB: species (Human, Mouse, Rat, Dog), application (WB, Flow cyto, ELISA, IHC-P, IF, ICC,), manufacturer's website (https://www.abcam.com/gfp-antibody-chip-grade-ab290.html)
LaminB (1:2,000; Santa Cruze; SC-6216) for WB: species (Human, Mouse, Rat), application (WB, IP, IF, IHC), manufacturer's website (http://datasheets.scbt.com/sc-6216.pdf)
ERK (1:2,000;BD Biosciences; 610408) for WB: species (Human, Mouse, Dog, Rat, Chicken, Frog), application (WB, IP, IF, IHC), manufacturer's website (http://www.bdbiosciences.com/us/applications/research/stem-cell-research/stem-cell-signaling/human/purified-mouse-anti-erk1-mk1/p/610408)

Animals and other organisms

Policy information about studies involving animals; ARRIVE guidelines recommended for reporting animal research

Laboratory animals

C57BL/6 male mice (WT, ZFP36L1+/-) were subjected to induction of experimental OA. ZFP36l1+/- mice (8-bp insertion/42-bp deletion in exon 2) were generated by ToolGen, Inc. Homozygous KO mice (Zfp36l1-/-) are not viable. We therefore used heterozygous Zfp36l1+/- mice for our experimental OA studies.
For primary cell culture, chondrocytes were isolated from the femoral condyles and tibial plateaus of postnatal day 5 WT mice.
Experimental OA was induced by DMM (destabilization of the medial meniscus) surgery or IA (intra-articular) knee injection.
12 week old male mice were used.

Wild animals

This study did not involve wild animals.

Field-collected samples

This study did not involve animals collected from the field.

Human research participants

Policy information about studies involving human research participants

Population characteristics

International Cartilage Repair Society (ICRS) grade 4 human knee cartilage tissues were obtained from aged 50-74 years old patients. The cartilage tissues offered by these patients were grouped by damaged and undamaged region for the following analysis. All recruited patients did not have rheumatoid arthritis, which disease can affect the cartilage and synovial membrane. Sex, weight and height of patients were not controlled.

Recruitment

Patients with a osteoarthritis undergoing arthroplasty were recruited for offering the cartilage tissue. Patients diagnosed with International Cartilage Repair Society (ICRS) grade 4 were eligible for cartilage harvesting and following analysis. Institutional Review Board of Wonkwang University Hospital approved the use of these tissues, and written informed consent was obtained from all patients before the operative procedure. Participants were recruited on an "as available" basis by participating surgeons (Churl-Hong Chun). Recruitment bias is unlikely to impact this study as no patient comparisons are performed and no analysis of demographic or clinical covariates on cellular properties is performed.
